# Natural Shorelines Promote the Stability of Fish Communities in an Urbanized Coastal System

**DOI:** 10.1371/journal.pone.0118580

**Published:** 2015-06-03

**Authors:** Steven B. Scyphers, Tarik C. Gouhier, Jonathan H. Grabowski, Michael W. Beck, John Mareska, Sean P. Powers

**Affiliations:** 1 Department of Marine and Environmental Sciences, Marine Science Center, Northeastern University, Nahant, Massachusetts 01905 United States of America; 2 The Nature Conservancy, Global Marine Program, Santa Cruz, California 95060 United States of America; 3 Alabama Department of Conservation and Natural Resources, Post Office Box 189, Dauphin Island, Alabama 36528 United States of America; 4 Department of Marine Sciences, University of South Alabama & Dauphin Island Sea Lab, Mobile, Alabama 36688 United States of America; University of Sydney, AUSTRALIA

## Abstract

Habitat loss and fragmentation are leading causes of species extinctions in terrestrial, aquatic and marine systems. Along coastlines, natural habitats support high biodiversity and valuable ecosystem services but are often replaced with engineered structures for coastal protection or erosion control. We coupled high-resolution shoreline condition data with an eleven-year time series of fish community structure to examine how coastal protection structures impact community stability. Our analyses revealed that the most stable fish communities were nearest natural shorelines. Structurally complex engineered shorelines appeared to promote greater stability than simpler alternatives as communities nearest vertical walls, which are among the most prevalent structures, were most dissimilar from natural shorelines and had the lowest stability. We conclude that conserving and restoring natural habitats is essential for promoting ecological stability. However, in scenarios when natural habitats are not viable, engineered landscapes designed to mimic the complexity of natural habitats may provide similar ecological functions.

## Introduction

Coastal habitats host diverse ecological communities and provide numerous ecosystem services that affect the health, security and quality of life of human societies [1,2]. The degradation or loss of natural habitats is a ubiquitous problem for urbanized coastal regions and results from a multitude of anthropogenic stressors such as shoreline development and pollution [3–5]. Conservation scientists have made substantial efforts to understand the consequences of habitat degradation or loss, and have shown that the potential for recovering lost ecosystem functions and services exists if natural habitats are sufficiently protected and restored [6]. However, in urbanized coastal settings, restoring natural landscapes to their historical baselines is unrealistic in part due to the prominence of artificial and engineered shorelines implemented for coastal protection and erosion control [5]. For instance, largely featureless seawalls and bulkheads can degrade natural habitats, and typically support less diverse ecological communities than vegetated shorelines [7,8]. Conversely, structurally complex artificial structures mitigate some of these negative ecological consequences of urbanization along shorelines [9,10]. However, it is still unclear how the increased prevalence of engineered shorelines affects ecological communities in urbanized ecosystems at broader scales.

The concepts of stability and resilience have been central foci of both fundamental ecology and applied conservation for at least the past half-century [11–14]. Stability and resilience are often characterized by the tendency of a system to fluctuate less [15,16] or its capacity to absorb perturbations and still maintain function [11,17]. However, the complexity of ecological communities and the inherent non-linearity of ecosystem functions complicate the study of resilience in dynamic ecosystems [18–20]. Studies that reveal critical properties that consistently promote the stability and resilience of communities exposed to heavy and dynamic disturbance regimes will contribute fundamentally to our understanding of how ecosystems function and help managers design strategies that ensure the maintenance of key ecosystem services.

The utilization of engineered coastal structures such as vertical walls and revetments directly replaces natural shoreline habitats, disrupts land-water exchange, and alters the biophysical environment (e.g., wave climate, depth profile), potentially indirectly harming other natural habitats [8,21,22]. Only recently, and largely in response to major disasters such as Hurricanes Katrina and Sandy, have coastal protection initiatives focused on incorporating ecological and ecosystem processes alongside physical and engineering objectives [2,23]. Moreover, it is essential that we understand how the growing number of engineered landscapes impact the structure and resilience of ecological communities, which in turn will impact the delivery of ecosystem services.

Although the societal and ecological costs of coastal habitat degradation are becoming increasingly recognized [23–25], coastal population size and development have continued to expand. However, very few studies to date have directly considered how the coastal protection structures that are currently replacing natural coastline features affect the stability or resilience of ecological communities [10,26], even though these communities when intact are highly productive and contribute to many valuable ecosystem services associated with coastal ecosystems. Here we couple high resolution shoreline condition data and an eleven-year time series of coastal fish abundances to examine how shoreline condition affects fish community stability and structure. We predicted that the communities associated with natural landscapes would fluctuate less than those near engineered shorelines, especially vertical walls that provide little to no habitat structure.

## Materials and Methods

### Ethics Statement

This study involves the analysis of data resulting from routine fisheries research and monitoring efforts by the State of Alabama Department of Conservation and Natural Resources—Marine Resources Division (ADCNR-MRD). ADCNR-MRD research and monitoring is regulated and permitted by the State of Alabama. ADCNR-MRD’s standard protocol for sampling vertebrate fishes involves the live release of specimens when possible. This study did not involve endangered or protected species. The data analyzed and interpreted in this study were collected in Mobile Bay, Alabama USA (30.460795, -87.993580).

### Description of Study System

**Fig 1:**
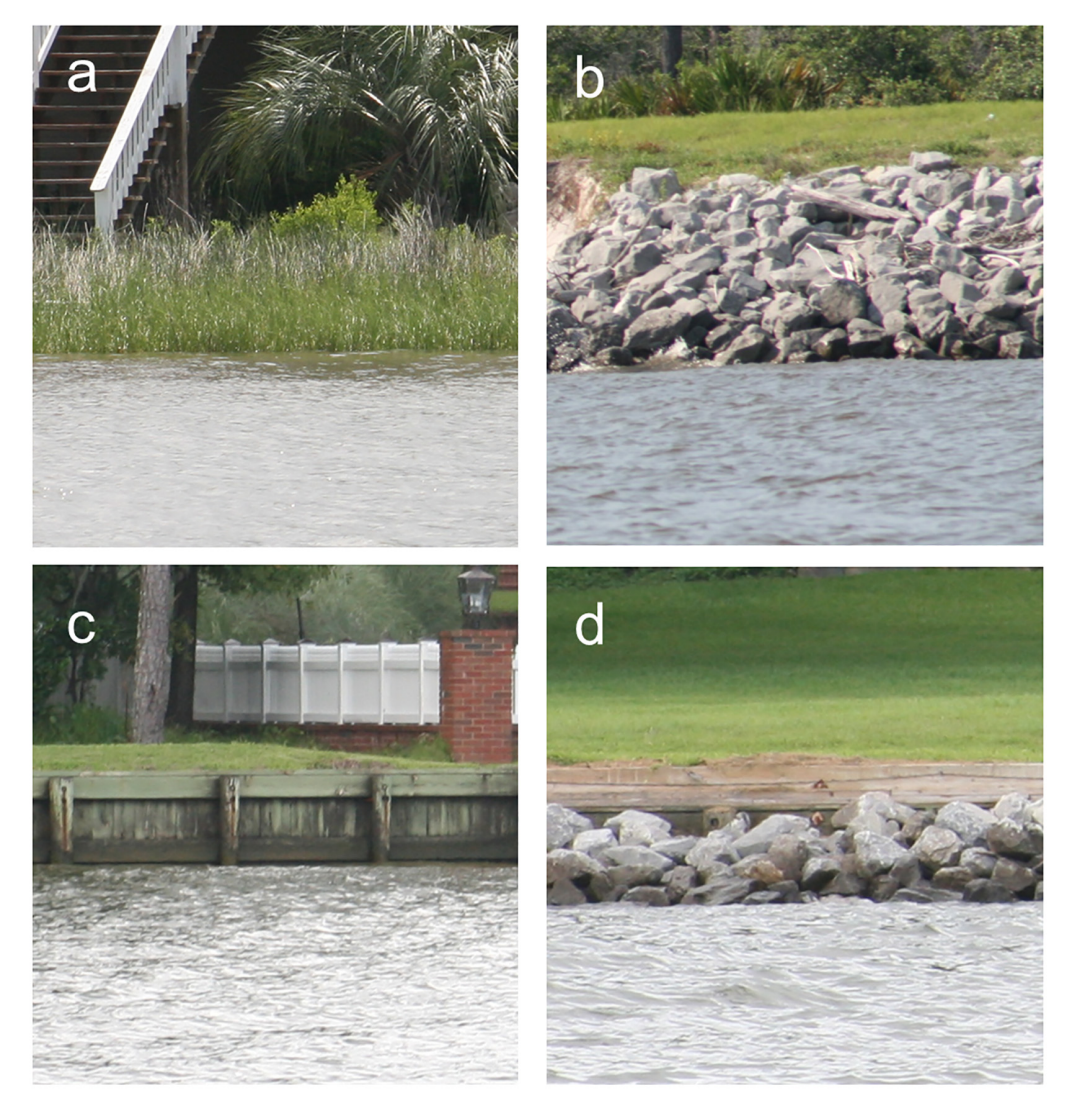
Example photographs of shoreline types. Photographs of (a) natural, (b) riprap / rubble, (c) vertical wall and (d) vertical wall with riprap shorelines in the study system.

Mobile Bay is a typical estuarine embayment located in the northern Gulf of Mexico and is an exemplary case of how coastal development and shoreline transformation are directly related to human population expansion [21]. Specifically, a recent geological assessment estimated that 38% of the bay’s shoreline had been transformed into engineered structures such as vertical walls and revetments (Fig 1), and approximately 93% of the shoreline was experiencing erosion [27]. From the pre-development conditions, it has been estimated that at least 5 to 10 hectares of intertidal habitat has been lost in this microtidal bay [21]. Our study encompassed 1,075 km^2^ of bay water surrounded by 235 km of shoreline (Figure A in S1 File).

### Data

We acquired high resolution data from a 2009 shoreline condition assessment by the Geological Survey of Alabama (GSA) [27]. The GSA shoreline protection data were developed through field surveys using a small vessel and real-time data recording to document materials and structures seaward, along, and landward of the shoreline. We reduced the 14 categories utilized for their classification scheme into the following four general categories which represent > 98% of the shoreline: 62% natural (e.g., vegetated), 10% riprap or rubble revetment, 17% vertical wall (e.g., bulkhead, seawall), and 10% vertical wall with riprap (Fig 1; Table A in S1 File).

Eleven years of monthly data on coastal fish communities (2001–2011) were acquired from a fishery-independent state survey designed to monitor juvenile and adult finfish populations. The survey was initiated in 2001 with a randomly stratified sampling scheme throughout five zones in the northern and southern regions the bay (Figure A in S1 File). The survey sampling design involves using small and large mesh experimental gillnets fished for one hour. The smaller net consists of 5 panels, each 45.7 m in length, with mesh sizes of 5.1, 6.4, 7.6, 8.9 and 10.2 cm stretched. The large mesh net consists of 4 panels, each 45.7 m in length, and mesh sizes of 11.4, 12.7, 14.0 and 15.2 cm stretched. Sampling location, time and net deployment configuration relative to the shoreline (parallel or perpendicular) were randomly assigned for each sampling event. During each sampling effort, environmental parameters of water temperature, salinity, dissolved oxygen, depth and tidal range were recorded. The gear-specific and monthly distribution of sampling effort is provided in S1.

### Analyses

We utilized univariate, multivariate and time-series analyses to assess how shoreline condition affects coastal fish communities. To perform these analyses, we combined the fish community and shoreline condition data by associating each gillnet sample with the closest shoreline condition. We explored temporal patterns of community similarity and environmental variability (temperature, salinity, depth, dissolved oxygen) across shoreline types using permutational multivariate analysis of variance (PERMANOVA) and non-metric multidimensional scaling (nMDS) analysis on Bray-Curtis distances [28]. To quantify ecological stability, we applied ANOVA on the Euclidean distance in ordination (nMDS) space between mean annual fish community structure observed in all pairs of years for each shoreline type. Finally, we utilized univariate wavelet analysis to document how the relative contribution of fluctuations at different frequencies or periods to the overall variance in the time series of total fish abundance and species richness changed over time for each shoreline type (see S2 for detailed methodology). Specifically, we used the global wavelet power spectrum, which represents the temporally-averaged variability at each period, to compare the temporal variance of average fish abundance and species richness associated with different shorelines at monthly to multi-annual periods from 2001 to 2011. To obtain the global wavelet power spectrum for average fish abundance for each shoreline, we first used the Morlet wavelet to decompose the total variance of each species’ abundance time series over the time and frequency domains. For each species, this mathematical decomposition yielded a wavelet power spectrum (i.e., variance of each species’ abundance as a function of time and frequency or period), which was then averaged over the duration of the study to produce the global wavelet power spectrum. The global wavelet power spectrum was subsequently averaged across all species to produce a measure of mean variability across the fish community associated with each shoreline type. The same approach was used to compute the mean variability of species richness associated with each shoreline type. All processing and analyses were conducted using R 3.0.1 [29] and the biwavelet package [30].

## Results

**Fig 2:**
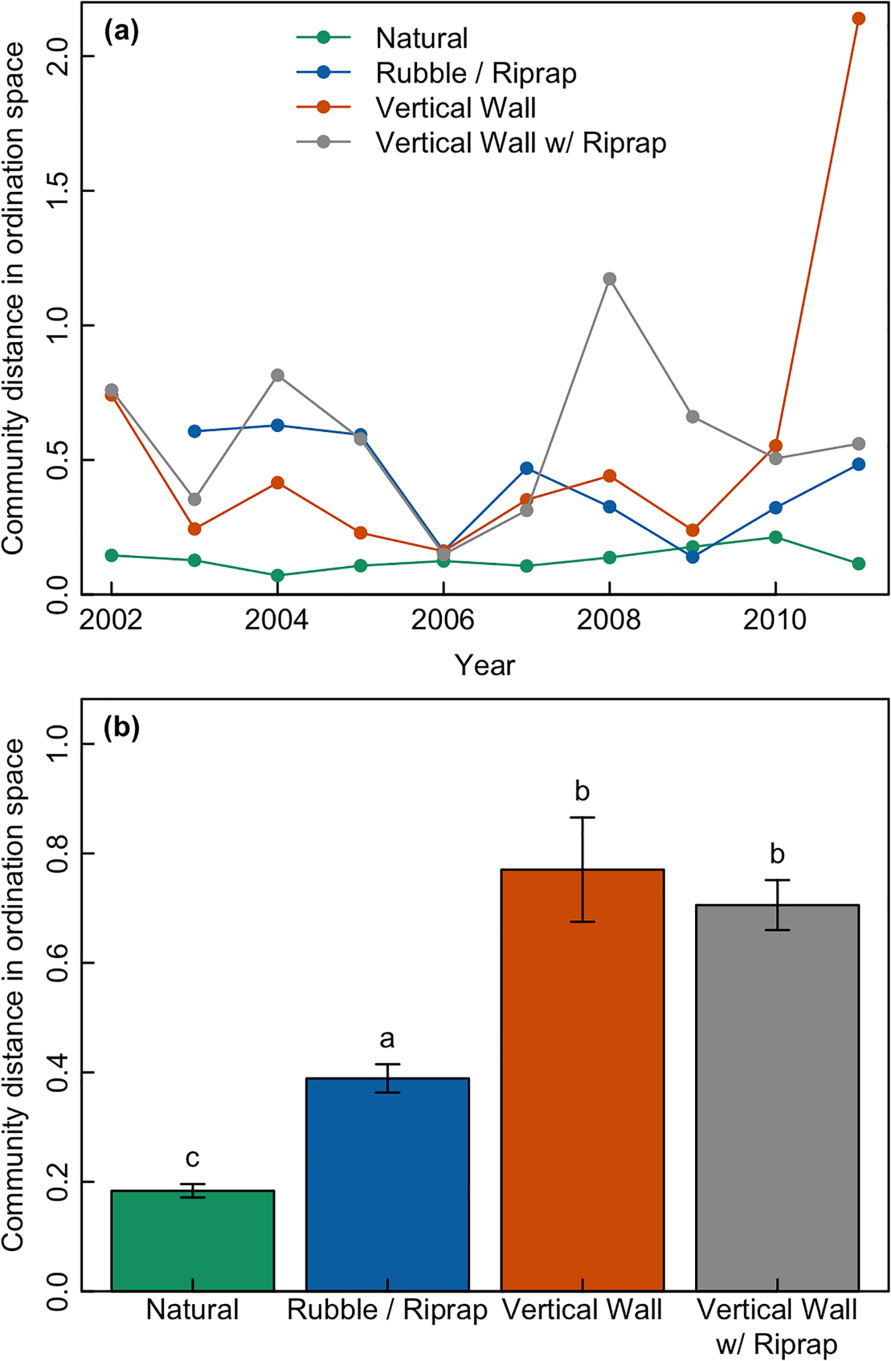
Fish community dissimilarity by shoreline type. Community dissimilarity measured by computing the Euclidean distance in (nMDS) ordination space between mean annual fish community structure observed in all pairs of years for each shoreline type (a). Mean Euclidian distance between fish community structure observed in all pairs of years for each shoreline type (+/- standard error of the mean) (b). Different letters indicate statistically significant differences based on ANOVA and post hoc pairwise comparisons using Tukey-Kramer’s HSD.

**Fig 3:**
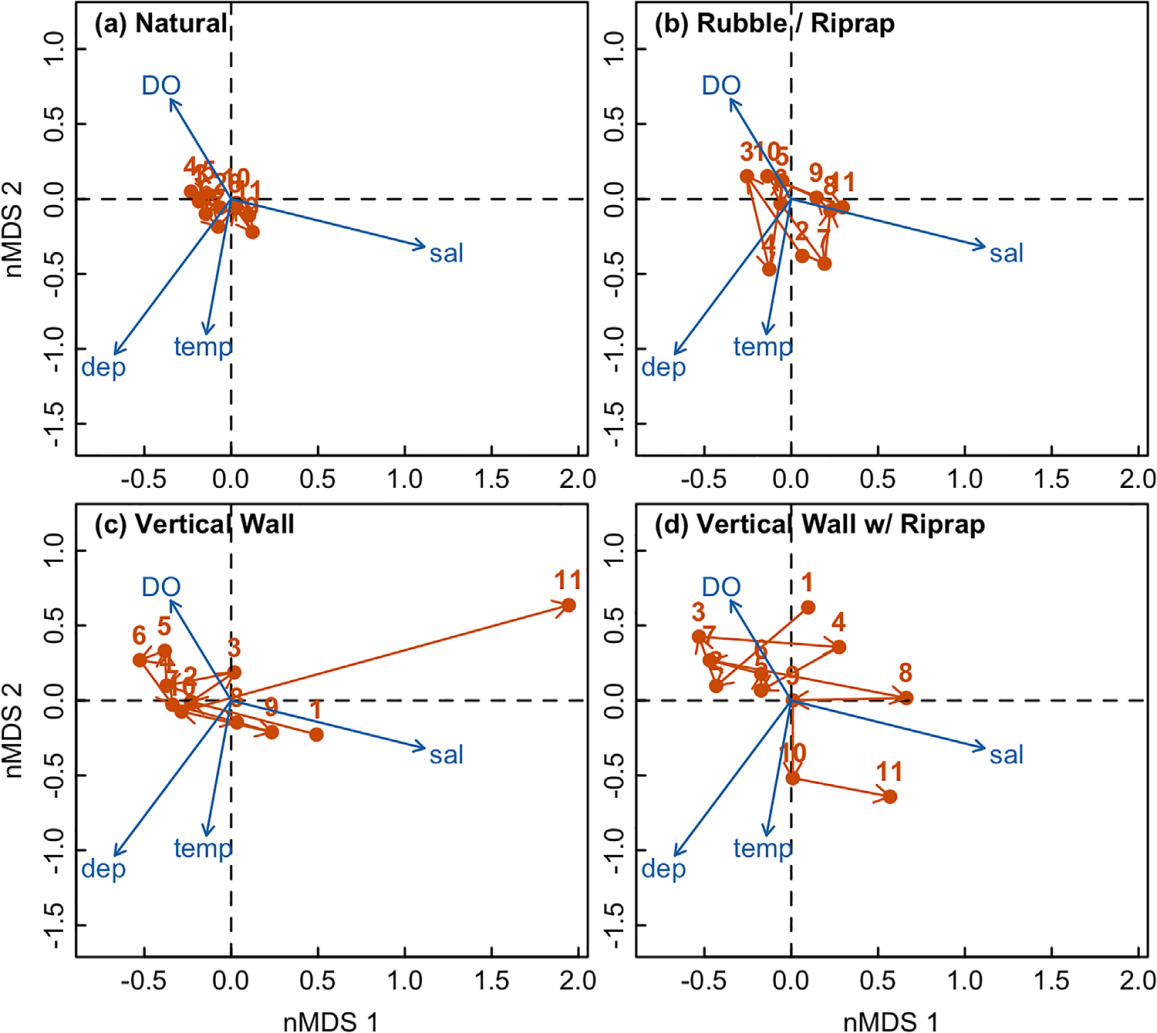
Non-metric multidimensional scaling (nMDS) plots for fish communities associated with each shoreline type. A single nMDS analysis was performed on the entire dataset (2-D stress = 0.13) and the results were plotted on four different panels based on shoreline type: (a) Natural, (b) Rubble with Riprap, (c) Vertical Wall, (d) Vertical Wall with Riprap. Blue vectors and labels indicate explanatory variables that are significantly correlated with the nMDS axes. Red arrows indicate the trajectory of communities over time, with the red labels representing year number.

Analysis of the Euclidean distance in ordination space between mean annual fish community structure observed in all pairs of years revealed that temporal variability of fish communities was related to shoreline condition (Table 1). Communities associated with natural shorelines varied less (i.e., had smaller Euclidean distances between communities measured in different years) than those associated with engineered landscapes (Fig 2). Similarly, PERMANOVA analysis of community similarity between successive years based on Bray-Curtis distance found that shoreline condition, sampling region, year and all measured environmental parameters affected fish community structure (Table 2). There were no significant interactions between factors. Plotting similarity based on Bray-Curtis distance for each shoreline condition with annual trajectories revealed higher temporal variability in communities associated with engineered shorelines (Fig 3).

**Table 1:** Results of ANOVA on the Euclidean distance in ordination space between mean annual fish community structure and shoreline condition.

	Df	SS	MS	F	P
**Shoreline type**	**3**	**11.419**	**3.806**	**49.839**	**< 0.0001**
Residuals	206	15.733	0.076		

**Table 2:** Results of PERMANOVA analysis of fish community structure.

	Df	SS	MS	F	R2	P
**Shoreline type**	**5**	**3.136**	**0.627**	**1.678**	**0.011**	**0.001**
**Temperature**	**1**	**10.099**	**10.099**	**27.021**	**0.036**	**0.001**
**Salinity**	**1**	**1.918**	**1.918**	**5.131**	**0.007**	**0.001**
**Dissolved Oxygen**	**1**	**0.701**	**0.701**	**1.875**	**0.002**	**0.018**
**Depth**	**1**	**3.100**	**3.100**	**8.295**	**0.011**	**0.001**
**Year**	**10**	**9.803**	**0.980**	**2.623**	**0.035**	**0.001**
**Region**	**1**	**0.858**	**0.858**	**2.295**	**0.003**	**0.004**
**Sub-region**	**8**	**5.128**	**0.641**	**1.715**	**0.018**	**0.001**
Residuals	665	248.549	0.374		0.877	
Total	693	283.291			1	

**Fig 4:**
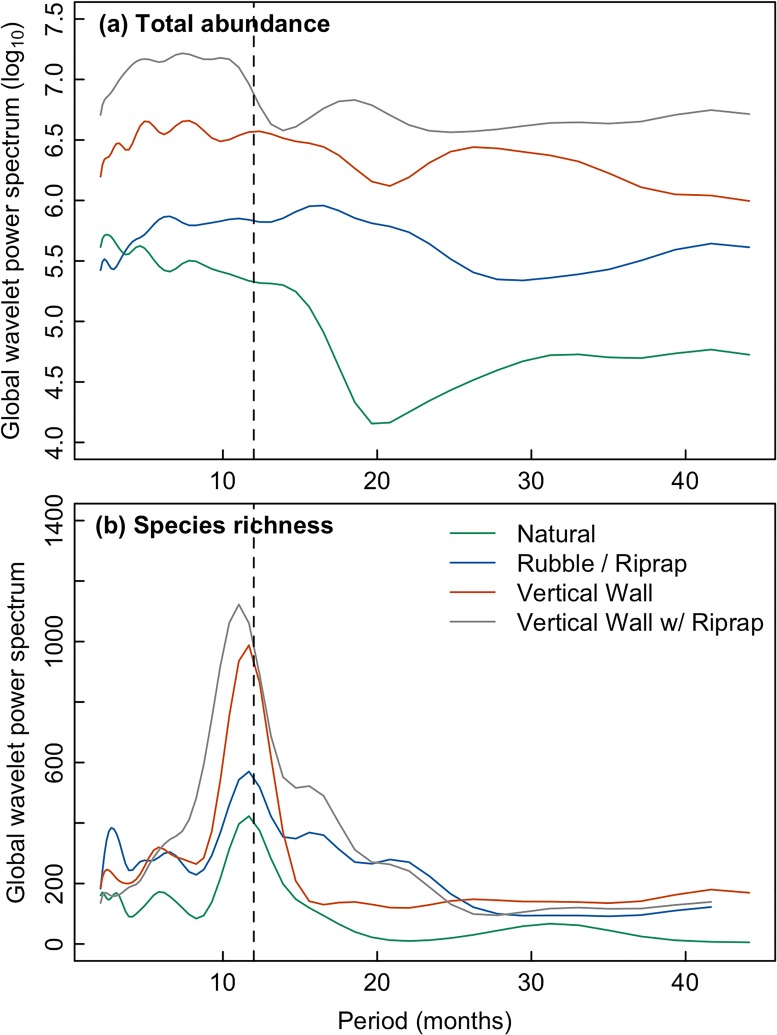
Global wavelet power spectrum of monthly average abundance and species richness by shoreline type. The global wavelet power represents the time-averaged variance of a signal (i.e., total abundance in panel [a] and species richness in panel [b]) at each period. For average abundance, wavelet analysis was performed for each species to obtain the global wavelet power spectrum. The global wavelet power spectrum was then averages across all species.

To determine whether these stability differences between shoreline conditions were present at sub- and super-annual periods, we computed the global wavelet power spectrum, which measures the average temporal variability at each period from 2001 to 2011. For average abundance, communities associated with natural shorelines had much lower temporal variance (i.e., greater stability) than that of the engineered shorelines at periods ranging from 2–40 months (Fig 4a). For species richness, global wavelet power revealed strong variability at annual scales (vertical dashed line) for fish communities associated with all shoreline conditions (Fig 4b). However, the global wavelet power remained systematically lower for natural shorelines than engineered shorelines in all periods (Fig 4b). Collectively, these results indicate that the overall differences in shoreline types determined by the nMDS plots, which represented the average annual variation in community structure, also apply to species richness and average abundance at monthly to multi-annual periods.

## Discussion

We found that natural landscapes support more stable fish communities than engineered landscapes. However, not all engineered landscapes performed identically. For instance, the stability of coastal fish communities was significantly higher near engineered shorelines characterized by rubble and riprap revetments than vertical walls or vertical walls with riprap. This finding suggests that structural complexity can in some instances reduce the negative effect of engineered structures on community stability. Natural habitats such as saltmarsh, oyster reef and submerged aquatic vegetation are structurally complex and widely recognized for providing essential habitat and nursery grounds for a variety of coastal species [31–34]. On the other hand, vertical walls typically provide very little structural complexity, and their presence often destroys proximal natural habitats by reflecting wave energy and enhances erosive processes on adjacent shorelines [7,21,35]. Since riprap revetments appeared to promote greater fish community stability than vertical walls, our study provides further evidence that structurally complex alternatives may be less ecologically harmful when shoreline armoring is deemed necessary [9,10].

By analyzing community similarity and variability at annual and monthly intervals, we assessed typical fluctuations or trends of stability and evaluated the potential impacts of discrete events of disturbance. For both the sequential and overall annual time series analyses, communities associated with natural shorelines exhibited higher community similarity and fluctuated less than all engineered shoreline conditions. The global wavelet power analyses indicated that the high resilience of natural landscapes, which was observed at annual timescales, also applies at shorter time periods. During the 11 years that were examined in our study, the Alabama Gulf coast was impacted by several hurricanes and tropical storms including Allison in 2001, Ivan in 2004, Dennis, Katrina and Rita in 2005 and Ike in 2008. The Gulf of Mexico also experienced a massive oil spill following the explosion of the Deepwater Horizon drilling rig in 2010. However, studies of tidal marsh creeks following Ivan and seagrass meadows following Katrina found very little impact of the hurricanes on coastal habitats [36,37]. The 2010 oil spill appears to have had no detectable immediate and direct effect on the coastal habitats of Mobile Bay, but the long term and indirect effects of response actions including precautionary fishing closures remain unclear [38]. Our findings indicate that fish communities adjacent to natural shorelines were resilient to disturbance from each of these stressors, and communities associated with engineered shorelines exhibited higher temporal variability.

Although engineered shorelines that mimic the complex structure of natural coastal habitats can partially restore community stability at local scales, preserving natural habitats may be important for community stability at both local and regional scales by “spilling over” via dispersal. Indeed, in fluctuating and interconnected metacommunities experiencing different environmental conditions (e.g., disturbance regimes, habitat types), connectivity can have a large impact on community stability across scales [39,40]. In the absence of connectivity, local communities will fluctuate asynchronously because of differences in local conditions. In such cases, local community stability will be low, but regional or metacommunity stability will be high because of the statistical averaging of asynchronously fluctuating local communities. Conversely, when connectivity is high, stability will be low at local and regional scales because dispersal will lead to large and synchronized fluctuations in community dynamics [39]. Hence, maintaining natural habitats and some level of connectivity may be critical for stability by supporting “spillover” into engineered habitats, and thereby promoting the persistence of the entire metacommunity. Determining the minimum level of connectivity and proportion of natural habitat required to promote stability without causing spatial synchrony is critical in order to preserve functions in increasingly altered ecosystems. Furthermore, understanding how the spatial extent and geographical distribution of natural habitats affect the sustainability of fisheries by controlling the delivery of larvae and adults into adjacent exploited ecosystems is critical for developing effective management programs [41–43].

The legacy and extraordinary degree of shoreline alteration in Mobile Bay, like many other coastal systems, dates back far longer than comprehensive ecological monitoring, making it quite challenging to understand how current fish communities adjacent to different shoreline types actually compare to a natural coastal community. However, the emergence of landscape ecology and the availability of longer term data series on ecosystem change have greatly improved our ability to understand how human activities have transformed the structure and function of natural landscapes [44–46]. For coastal ecosystems, these transformations have almost exclusively resulted in less desirable ecological conditions such as declining fisheries and water quality [6,47]. The transformation of coastal shorelines with artificial and engineered structures has been occurring for centuries but has rapidly increased in recent decades in part due to growing coastal populations and the cascading consequences of increasingly urbanized coastal ecosystems [25]. Further ecological studies in these increasingly urbanized settings are needed to resolve many uncertainties regarding the processes that mediate spatial and temporal variability in the habitat functioning of natural and engineered coastlines. Only recently have the potential impacts of coastal protection structures on ecosystems or human well-being been considered [10,18,23,26]. Our results indicate that conserving and restoring the integrity of natural habitats is the best approach for enhancing the resilience of coastal fish communities. In heavily developed systems and other settings where natural habitats may no longer be viable, our findings indicate that requiring coastal protection schemes provide structural complexity may mitigate some of the ecological impacts of coastal development. However, such structurally complex coastal protection features may only mimic this one function of natural habitats, and may not compensate for the loss of other ecosystem functions when natural habitats are degraded.

## Supporting Information

S1 File Map, description of gear-specific and seasonal distribution of sampling effort, and classification scheme for shoreline condition categories. (DOCX)

S2 File Wavelet analysis of total abundance and species richness for fish communities associated with different shoreline types. (DOCX)
