# Supplementary material for: Natural Shorelines Promote the Stability of Fish Communities in an Urbanized Coastal System
Source: PLoS One. 2015 Jun 3;10(6):e0118580. doi: 10.1371/journal.pone.0118580 (PMC4454662; doi:10.1371/journal.pone.0118580)
Supplement: S1 File — (DOCX) [file pone.0118580.s001.docx]

**File S1**: Map and description of sampling effort.

**Figure A**: Map of sampling locations and shoreline condition in Mobile Bay, Alabama.

**
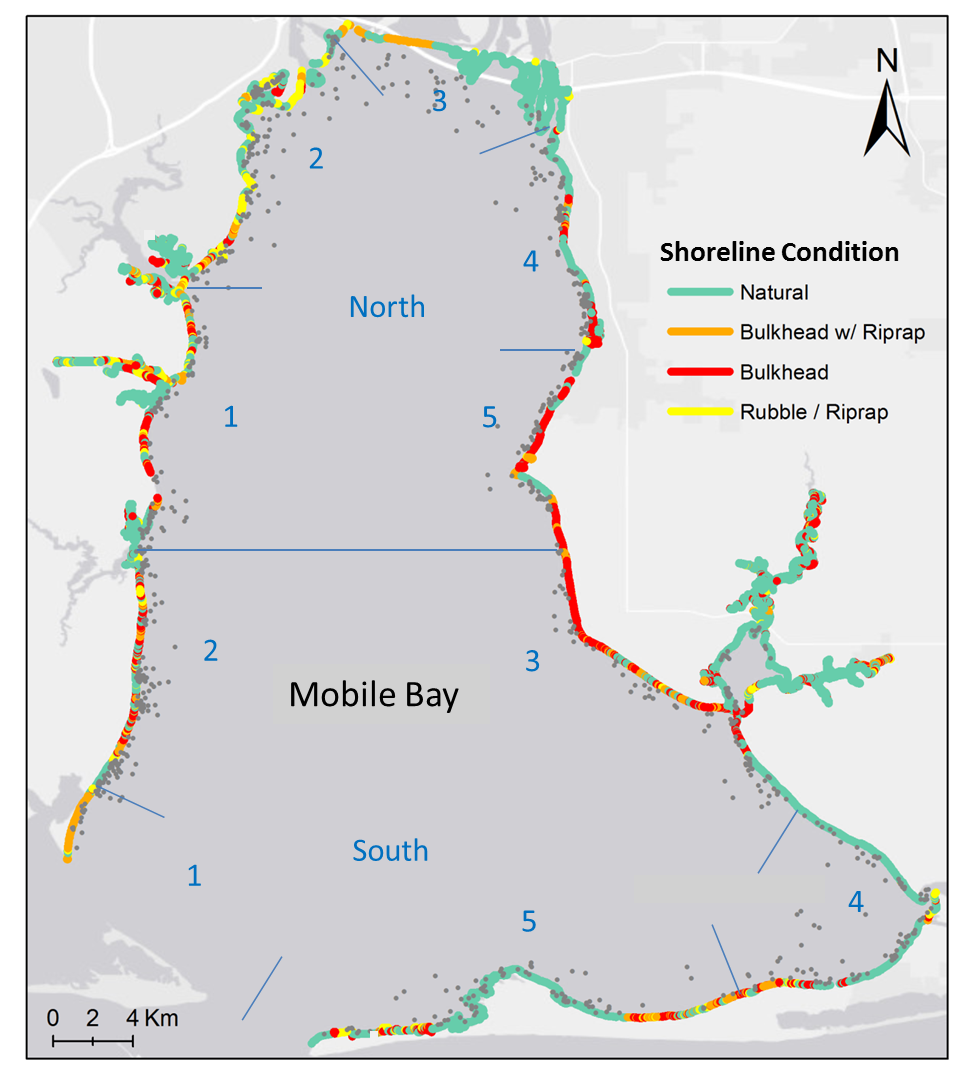
**

**Table A**: Shoreline classification scheme from Jones 2009 and the corresponding categories used in our analyses.

| **Vertical Walls** | **Vertical Walls w/ Riprap** | **Natural** | **Riprap Revetment** | **Excluded** |
| --- | --- | --- | --- | --- |
| Bulkhead (steel, wood) | Bulkhead (concrete, rock with riprap) | Beach Nourishment | Breakwater (offshore) | Abutment |
| Bulkhead (with groin) | Bulkhead (concrete, rock) | Bioengineered (vegetated) | Jetty (steel pile, rock, concrete) | Boat Ramp |
| Bulkhead (with retaining walls) | Bulkhead (concrete with riprap) | Natural | Revetment |  |
| Bulkhead (with retaining walls and groin) | Bulkhead (with retaining walls and riprap) |  | Rubble/riprap |  |
| Bulkhead (with sill) | Bulkhead (with riprap and groin) |  | Rubble/riprap (with groin) |  |
| Seawall (concrete, steel piles) | Bulkhead (with riprap) |  | Rubble/riprap (with tires) |  |
| Sill (wood) | Bulkhead (with riprap and sill) |  | Sill (rock, shell) |  |
| Groin | Bulkhead (with riprap, sill and groin) |  |  |  |

**Table B**: Monthly and gear-specific sampling effort for the Alabama Department of Natural Resources – Marine Resource Division gillnet fish survey.

| **Month** | **Jan.** | **Feb.** | **Mar.** | **Apr.** | **May** | **Jun.** | **Jul.** | **Aug.** | **Sep.** | **Oct.** | **Nov.** | **Dec.** |
| --- | --- | --- | --- | --- | --- | --- | --- | --- | --- | --- | --- | --- |
| **Small Mesh Effort** | 8 | 8 | 8 | 10 | 11 | 12 | 10 | 13 | 9 | 13 | 10 | 8 |
| **Large Mesh Effort** | 8 | 8 | 8 | 10 | 11 | 12 | 10 | 13 | 9 | 13 | 10 | 8 |
