# Supplementary material for: Natural Shorelines Promote the Stability of Fish Communities in an Urbanized Coastal System
Source: PLoS One. 2015 Jun 3;10(6):e0118580. doi: 10.1371/journal.pone.0118580 (PMC4454662; doi:10.1371/journal.pone.0118580)

**File S2**: Wavelet analysis of total abundance and species richness for fish communities associated with different shoreline types.

We performed wavelet analysis to determine how temporal fluctuations in total abundance and species richness varied in fish communities associated with different types of shorelines. We now provide a summary of these methods and direct the reader to previously published guides for greater details (e.g., Torrence and Compo 1998, Cazelles et al. 2008, Iles et al. 2012). Wavelets are flexible functions that are resolved in the time and frequency domains, and thus ideal for quantifying changes in the contribution of each period (or frequency) over time to the overall variance (or power) of a signal (e.g., a time series). Because wavelets can be scaled (i.e., contracted or dilated), they can efficiently accommodate both high and low frequency structures in non-stationary signals whose statistical characteristics vary over time. For our analyses, we used the Morlet wavelet, which is defined as:

where is the imaginary number, represents nondimensional time, and is the nondimensional frequency (Torrence and Compo 1998). The continuous wavelet transform of a discrete time series with equal spacing and length is defined as the convolution of with a normalized Morlet wavelet (Torrence and Compo 1998, Grinsted et al. 2004):

where * indicates the complex conjugate. By varying the wavelet scale (i.e., dilating and contracting the wavelet) and translating along localized time position , one can calculate the wavelet coefficients , which describe the contribution of the scales to the time series at different time positions (Torrence and Compo 1998, Cazelles et al. 2008). Here, is a parameter used to normalize the Morlet wavelet function to unit variance in order to allow direct comparisons of the wavelet coefficients across the different scales and time positions (Torrence and Compo 1998, Grinsted et al. 2004). Contour plots can be used to visualize how the local wavelet power spectrum (i.e., the contribution of each frequency or period in the time series) varies in time (Grinsted et al. 2004, Cazelles et al. 2008).

Additionally, one can compute the bias-corrected global wavelet spectrum as the time-average (i.e., over all time locations ) of all local wavelet spectra for each scale (Torrence and Compo 1998, Liu et al. 2007, Cazelles et al. 2008):

where represents the variance of the time series and is the bias correction factor (Liu et al. 2007). The global wavelet spectrum thus quantifies the average power (or variance) of the time series at each scale. The scale of the Morlet wavelet is related to Fourier frequency (Maraun and Kurths 2004, Cazelles et al. 2008): . When , the scale is approximately equal to the reciprocal of the Fourier frequency (Maraun and Kurths 2004, Cazelles et al. 2008): . Hence, in all equations, the scale can be converted to Fourier frequency or period .

### Zero-padding and the cone of influence

In practice, the continuous wavelet transform is computed by using discrete Fourier transforms to calculate all convolutions simultaneously (Torrence and Compo 1998). However, since the Fourier transform assumes that the data is periodic, errors in the estimation of the local wavelet power spectrum will occur at the beginning and at the end of any finite-length time series (Torrence and Compo 1998, Cazelles et al. 2008). In order to limit these edge effects, the end of a time series is padded with zeros prior to taking the wavelet transform and the zeroes are then removed (Torrence and Compo 1998, Cazelles et al. 2008). Typically, enough zeros are added in order for the total length of the time series to reach the next-higher power of two. This both limits edge effects and improves the speed of the Fourier transform (Torrence and Compo 1998).

Although padding with zeros limits errors due to edge effects, it introduces artificial discontinuities at the endpoints of the data (Torrence and Compo 1998, Cazelles et al. 2008). As one gets closer to the end of the data, more zeros are included in the estimation of the local wavelet spectrum, thus reducing its reliability (Torrence and Compo 1998, Cazelles et al. 2008). The region where zero padding affects the estimation of the wavelet spectrum is called the cone of influence (COI), and is defined as the region in which the wavelet power for a discontinuity at the edge drops by a factor of (Torrence and Compo 1998). Hence, any region falling below the COI is susceptible to edge effects.

### Statistical significance testing

In order to determine the statistical significance of the wavelet spectrum obtained from a time series, one must first formulate an appropriate null hypothesis. Here, the null hypothesis is that the observed time series is generated by a stationary process with a given background power spectrum (Torrence and Compo 1998, Grinsted et al. 2004). Since many ecological and environmental time series exhibit strong temporal autocorrelation (i.e. high power associated with low frequencies; e.g. Beninca et al. 2009, see Ruokolainen et al. 2009 for review), we used a first order autoregressive model [AR(1)] to generate a temporally autocorrelated time series or red noise, which served as our null hypothesis. Specifically, the power spectrum of our red noise process was calculated with (Gilman et al. 1963):

where the autocorrelation coefficient at time lag 1 is estimated from the observed time series and represents the frequency index. The observed wavelet spectrum can be compared to the wavelet spectrum of the red noise process by means of a chi-square test. The distribution of the local wavelet power spectrum of a red noise process is (Torrence and Compo 1998):

where represents the frequency index, represents the variance of the time series, “” means “is distributed as”, and represents the chi-square distribution with 2 degrees of freedom. The value of is the mean wavelet power spectrum at frequency that corresponds to the wavelet scale (Torrence and Compo 1998). Using this equation, one can construct 95% confidence contour lines at each scale using the 95^th^ percentile of the chi-square distribution (Torrence and Compo 1998).

**Literature Cited**

Beninca, E., K. D. Johnk, R. Heerkloss, and J. Huisman. 2009. Coupled predator-prey oscillations in a chaotic food web. Ecology Letters 12:1–12.

Cazelles, B., M. Chavez, D. Berteaux, F. Menard, J. O. Vik, S. Jenouvrier, and N. C. Stenseth. 2008. Wavelet analysis of ecological time series. Oecologia 156:287–304.

Gilman, D. L., F. J. Fuglister, and J. M. Mitchell. 1963. On the Power Spectrum of “Red Noise”. Journal of the Atmospheric Sciences 20:182–184.

Grinsted, A., J. C. Moore, and S. Jevrejeva. 2004. Application of the cross wavelet transform and wavelet coherence to geophysical time series. Nonlinear Processes in Geophysics 11:561–566.

Iles, A. C., T. C. Gouhier, B. A. Menge, J. S. Stewart, A. J. Haupt, and M. C. Lynch. 2012. Climate-driven trends and ecological implications of event-scale upwelling in the California Current System. Global Change Biology 18:783–796.

Legendre, L., and P. Legendre. 1998. Numerical Ecology, 2nd edition. Elsevier Science, Amsterdam.

Liu, Y., X. San Liang, and R. H. Weisberg. 2007. Rectification of the Bias in the Wavelet Power Spectrum. Journal of Atmospheric and Oceanic Technology 24:2093–2102.

Maraun, D., and J. Kurths. 2004. Cross wavelet analysis: significance testing and pitfalls. Nonlin. Processes Geophys. 11:505–514.

Ruokolainen, L., A. Lindén, V. Kaitala, and M. S. Fowler. 2009. Ecological and evolutionary dynamics under coloured environmental variation. Trends in Ecology & Evolution 24:555–563.

Torrence, C., and G. P. Compo. 1998. A Practical Guide to Wavelet Analysis. Bulletin of the American Meteorological Society 79:61–78.

**Figure legends**

**Figure A**: Wavelet analysis of monthly time series of total fish abundance in communities associated with shorelines characterized as (a) natural, (b) rubble with riprap, (c) vertical wall, and (d) vertical wall with riprap. Regions of high (low) power or variability are represented in warm (cold) colors. Black contours represent regions of statistically significant variability at the α=0.05 level. Regions within the white dashed lines (the cone of influence) are not affected by edge effects.

**Figure B**: Wavelet analysis of monthly time series of species richness in communities associated with shorelines characterized as (a) natural, (b) rubble with riprap, (c) vertical wall, and (d) vertical wall with riprap. Regions of high (low) power or variability are represented in warm (cold) colors. Black contours represent regions of statistically significant variability at the α=0.05 level. Regions within the white dashed lines (the cone of influence) are not affected by edge effects.

**Figure A**
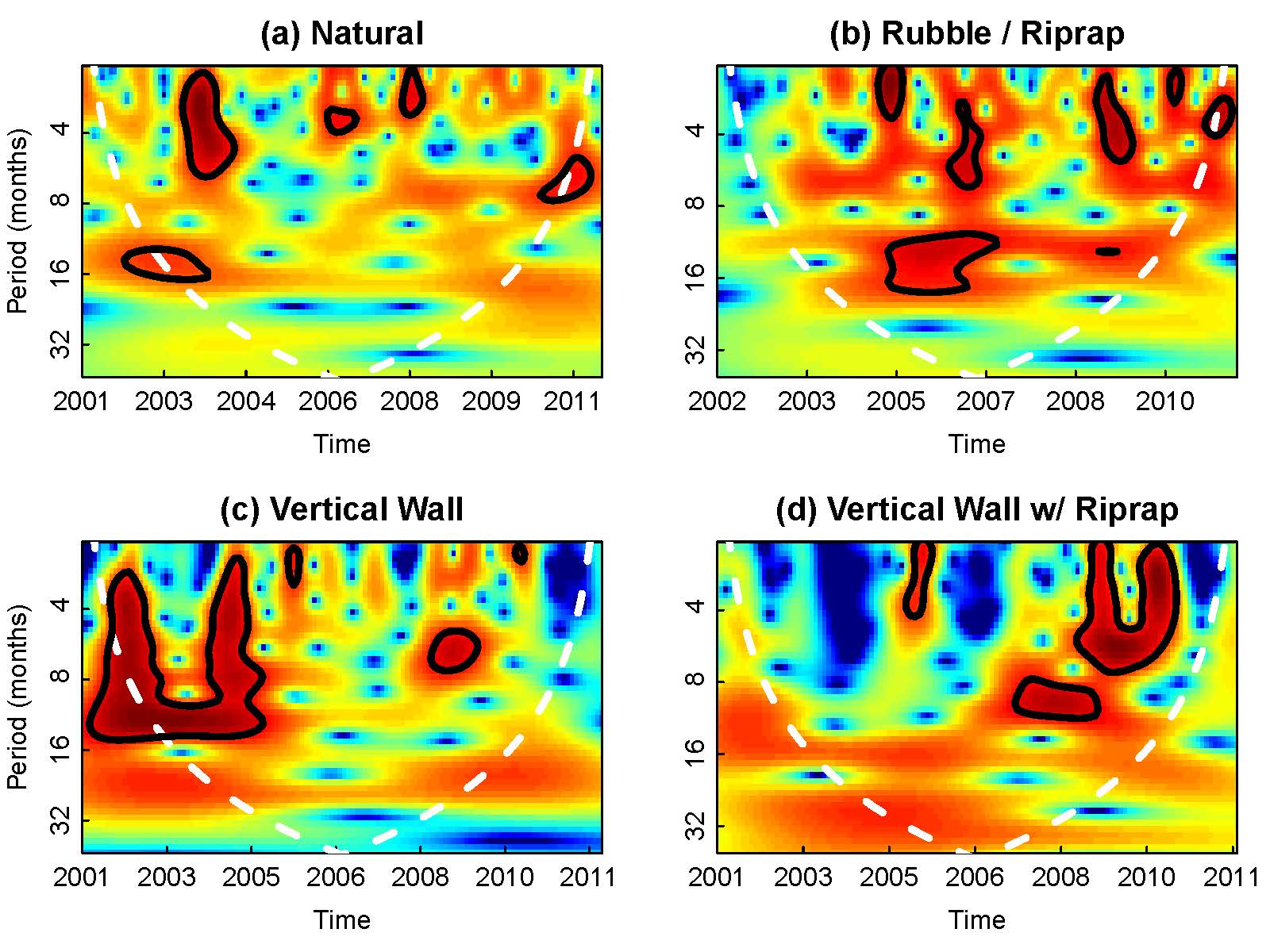


**Figure B**


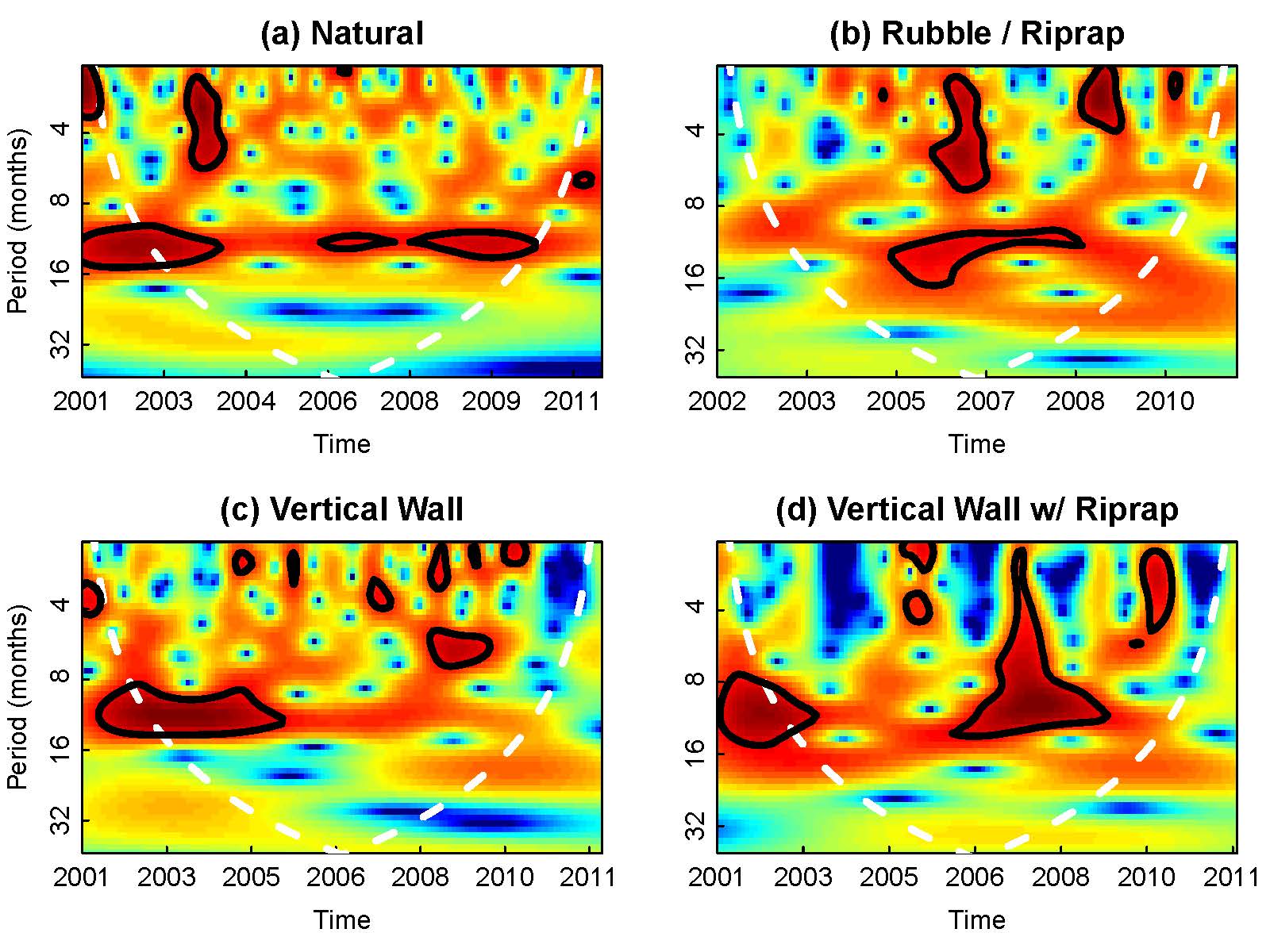

Supplement: S2 File — (DOCX) [file pone.0118580.s002.docx]
